# Effects of different patterns of maize-straw application on soil microorganisms, enzyme activities, and grain yield

**DOI:** 10.1080/21655979.2021.1931639

**Published:** 2021-07-13

**Authors:** Xilin Ning, Xiaohui Wang, Zheyun Guan, Yan Gu, Chunsheng Wu, Wenhe Hu

**Affiliations:** a Jilin Agricultural University, Changchun, China; b Jilin Academy of Agricultural Sciences, Changchun, China

**Keywords:** Microorganisms, phospholipid fatty acid, soil enzyme

## Abstract

The present study aimed to assess the influences of corn straw application on the soil microbial organisms, soil enzyme activities and the grain yield. Four treatments were evaluated: (i) The straw was ploughed into soil using a fence hydraulic turning plow with ploughing depth of 30-40 cm(PD). (ii) The self-developed straw deep returning machine was used to bury 30-40 cm in the sub-surface layer of soil (SD). (iii) The straw was mulched and no tillage sowing(M). (iv)Without straw application(CK). Soil samples of different deep(0-20 cm, 20-40 cm soil layer) were taken during the corn growth stage to determinesoil biological characteristics.Our results suggested that soil microorganisms were not increased by straw mulching. Straw deep ploughing and returning (PD treatment) could effectively improve the phospholipid fatty acids(PLFAs) of bacteria, actinomycetes, and fungi, the activities of urease,invertase,dehydrogenase and polyphenoloxidase, even the grain yield. In 20-40 cm subsoil layer, the effects were more obvious than those of topsoil. The mean yield of PD treatment was higher than SD,M and CK. The results showed that the PLFA signatures and soil enzyme were both sensitive to the changes of soil environment condition by the application of straw. In the actual field production, we should adopt the appropriate way of straw returning to the field to achieve not only the improvement of soil quality, but also the increase of grain yield.

## Graphical abstract

**Figure:**
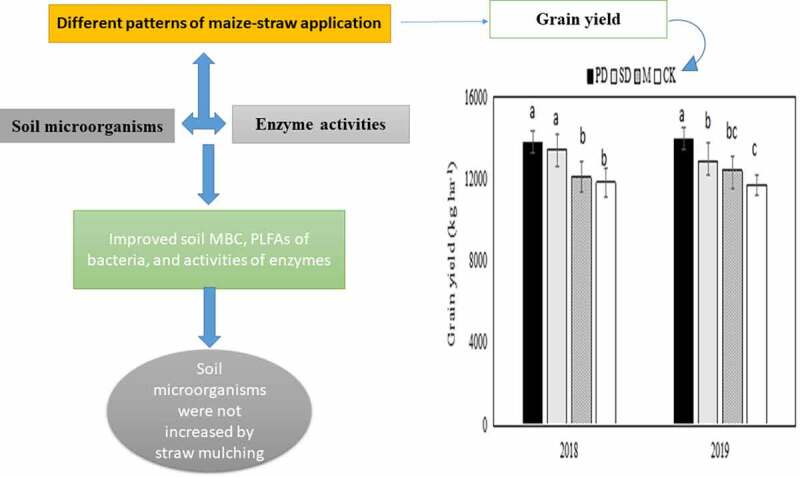

## Introduction

Jilin Province is located in northeastern China (40°52´–46°18´N, 121°38´–131°19´E) in one of the largest golden maize zones in the world and constitutes the commercial grain base of China. In 2017, the maize cultivation area accounted for 64.2% of the total grain area and 75.3% of the total production (Statistical Yearbook. Jilin University press. Bureau of Statistics in Jilin Province, 2017), with production predominantly occurring in the central humid and semi-humid rain-fed regions. The western part of Jilin Province is characteristic of its representative arid-semiarid zone featured by the little rain whereas great soil evaporation. In the past few years, people had applied a lot of chemical fertilizers to pursue the high yield, which had also caused a certain burden on the soil environment, and the soil quality has gradually degraded.

Straw incorporation has been considered to be the eco-friendly method to use straw byproducts, since it contains a large amount of nutrients [1]. As demonstrated in a number of studies, straw incorporation in fields can enhance the soil characters, thereby boosting soil moisture content [2], soil respiration [3], activity of soil enzymes [4–6], soil organic carbon (SOC) content, as well as soil fertility [7–9], along with the crop production [10]. The effects of different maize straw incorporation modes in the soil differ because various applications can affect different biogeochemical processes in soil, such as soil microbial biomass carbon, microbial community structure, and enzyme activities [11]. The soil microorganisms play an important role in maintaining crop productivity and the regulation of carbon fluxes, including plant decomposition and carbon sequestration in soil. Soil microbial biomass, microbial community, and enzyme activities are sensitive indicators of soil quality and processes [12,13].

Straw is widely used as a typical organic material in China. The burning of waste straw has become a concern worldwide, as it produces carbon dioxide, and other greenhouse gases. As such, straw incorporation in fields has been considered as beneficial by improving the crop productivity and soil fertility. The majority of studies have found discernable changes in soil microbial communities and enzyme activities following the straw layer burial or mulch in the field [14,15]. In the west of Jilin Province, local farmers have a variety of ways to return straw to the field. This study selected chernozem soil to study the influence of different straw application modes (straw buried into soil, self-developed straw deep in the sub-surface layer of soil and straw mulched without tillage sowing) on soil microbial compositions during the entire growth phase. The present study was, therefore, conducted to investigate the effects of straw application on soil microbial biomass carbon (C), microbial community structure, and enzyme activities. We aimed at identifying whether different patterns of maize-straw application have significant impact on soil microorganisms, or change the activities of soil enzymes, and determine whether there is a significant difference in grain yield.

## Materials and methods

### Site description

**Figure 1.**
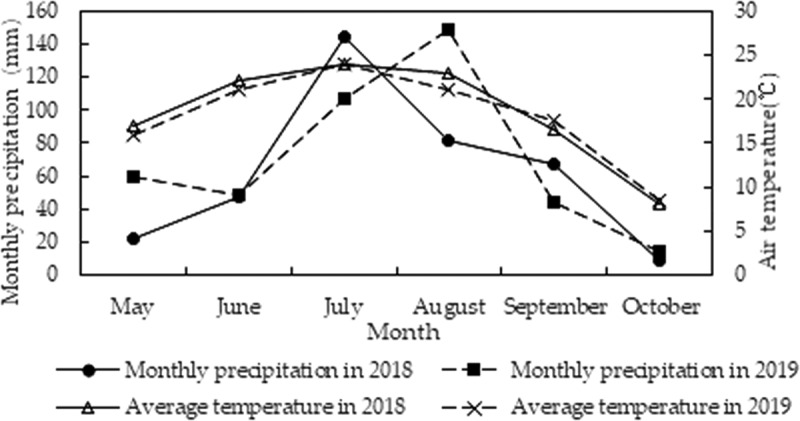
Average temperature and precipitation from May to October of 2018 and 2019 in the western area of Jilin province, China (data from China Meteorological Administration)

Field trials were carried out at the Western Area (124.8´, 45.17´) of Jilin province, China in 2018 and 2019. This region is characterized by a semiarid climate, while the average monthly temperature and rainfall during crop growth period are shown in Figure 1. The soil of the test site was categorized into the chernozem according to the Food and Agriculture Organization (FAO) classification, the organic matter content in the 0–20 cm soil layer was 1.40%, the total nitrogen content was 2.133 g/kg, the total phosphorus content was 353.83 mg/kg, and the content of alkaline hydrolysis nitrogen, available phosphorus, and available potassium was 75.91 mg/kg, 16.31 mg/kg, and 130.24 mg/kg, respectively, and the soil pH was 7.24.

### Experimental design

The tested maize variety, Hengdan 188 (Jilin Province Hengchang Agricultural Development Co., Ltd.) was cultivated under wide-narrow-row pattern (the ridge height was 12 cm; the upper row spacing was 50 cm; the row spacing between the ridges was 90 cm). After the corn was harvested on 4 October 2017, the stalks were crushed (the length < 10 cm) after harvest and scattered evenly in the field. The field was divided into four areas for different treatments of straw application, each with 8 ha. Four patterns of straw application were as follows: (i) The straw was plowed into soil using a fence hydraulic turning plow (1LYFT-450, Longfeng, China) with a plowing depth of 30–40 cm, and the machine was used for leveling the soil to reach the sowing state. (ii) The self-developed straw deep returning machine was used to bury 30–40 cm in the sub-surface layer of soil, and the order of the soil layer remained unchanged and no tillage sowing. (iii) The straw was mulched and no tillage sowing. (iv) Without straw application as the control. The above four treatments were referred to as PD, BD, M, and CK, respectively.

The field was sown on the 28^th^ of April and 26^th^ of April in 2018 and 2019, respectively. The planting density in all the plots was 75,000 plants ha^−1^ and the total fertilizer was as follows: N 280 kg/hm^2^, P 120 kg/hm^2^, and K 150 kg/hm^2^ each year. A base fertilizer included 30% of the total nitrogen fertilizer, all the phosphorus, and potassium fertilizer. Only 40% N and 30% N was applied at the 45 days and 65 days after maize planting with the dripping irrigation, respectively, in both years, and the rest of the N fertilizer was applied as a top dressing. The harvest date was September 26^th^, 2018, and October 3^th^, 2019.

### Soil sampling

At the different stages, V3 (3^th^ leaf, 25 May), V6 (6^th^ leaf, 15 June), V12 (12^th^ leaf, 10 July), VT (tasseling, 1 August), R3 (milk stage, 30 August), and R6 (physiological maturity, 25 September) [16], soil samples were collected from two layers (0–20 cm and 20–40 cm) at random five locations in each plot with a pipe 1 cm in diameter. The samples used for phospholipid fatty acid (PLFA) measurement were stored at −80°C and the remaining soil was used for the determination of soil microbial biomass carbon content (MBOC) and enzyme activities.

### Microbial biomass carbon

Chloroform fumigation-extraction approach [17] was utilized to determine soil microbial biomass C (MBC) level. First of all, fresh soil sample that was equal to 25 g dry soil was subjected to fumigation by chloroform (CHCl_3_) without ethanol under 25°C; at 24 h later, the chloroform was eliminated and the 0.5 M potassium sulfate (K_2_SO_4_, 100 mL) was added into the above soil sample to extract under 200 rev/min horizontal shaking for 30 min, and the sample was then filtered. Also, the 0.5 M K_2_SO_4_ (100 mL) was also used to extract the equivalent volume of the non-fumigated soil sample. Thereafter, the Total Organic Carbon Analyzer (Multi N/C 310 TOC, Germany) was utilized to determine the SOC level in each soil extract. Typically, MBC was determined by *E_C_/k_EC_*, where EC represents the difference in SOC level between fumigated soil extract and the non-fumigated counterpart, while k_EC_ equals 0.45.

### Phospholipid fatty acids (PLFA) analysis

[18], PLFA combination approach was utilized to determine the compositions of microbial communities. The chloroform:methanol:phosphate buffer solution [1:2:0.8, v/v] was utilized for PLFA extraction from the freeze-dried soils, later, the silica-bonded phase column was used for separation, and low alkaline methanolysis was adopted for trans-esterification of fatty acid methyl esters (FAMES). The FAMES were quantified with a gas chromatograph (Agilent N6890, Agilent Technologies, Santa Clara, CA, US) and were identified with a flame ionization detector [Palo Alto, CA, USA] of the method of [19]. Each PLFAs content was measured according to 19:0 internal reference levels per nmol/g dry soil. For all samples, their abundance values were expressed as the % mol abundance.

In all the identified fatty acids, 15:0, 16:0, 17:0, a15:0, a17:0, i15:0, i16:0, i17:0,16:1w7c, cy17:0, cy19:0 and 18:1w7c, were selected as bacterial biomarkers [20, 21]. The methylic mid-chain-branched saturated PLFAs 10Me16:, 10Me17:0, and 10Me18:0 were selected as indicators of actinomycetes [22], and 18:1w9c was used as a fungal biomarker [23]. The ratio of bacteria and fungi was determined.

### Enzyme activity

Soil enzyme activities (urease, invertase, dehydrogenase and polyphenoloxidase) were followed by [24]. Urea was used as a substrate to determine the urease [EC 3.5.1.5] activity. The soil mixture was incubated at 37°C for 5 h, and then was added to 50 mL of 1 M KCl solution and shaken for 30 min. The ammonium released from urea hydrolysis was determined with a UV spectrometer at 690 nm. A sucrose solution was used as a substrate to determine invertase [EC 3.2.1.26] activities. Soil was incubated at 37°C for 24 h in 15 mL 8% sucrose, 5 mL 0.2 M phosphate buffer, and 0.05 mL toluene. The mixture was filtered and added to 3,5-dinitrylsalicylate and 3-aminonitrosalicylic acid after incubation and then measured at 508 nm. The activities of urease and invertase were expressed as units of mg NH^4+^-N per 100 g soil within 24 h and mg glucose per g dry soil within 24 h, respectively.

[25], 2,3,5-triphenyltetrazolium chloride [TTC] approach after slight modification was used to determine the activity of dehydrogenase. Briefly, 6 g soil was blended completely with 60 mg calcium carbonate (CaCO_3_), while the mixture was later added in 3 test tubes (1.6 × 15 cm), followed by the addition of 3% TTC (1 mL) as well as deionized water (2.5 mL). Later, each sample was blended into the vortex mixer to incubate for 24 h under 37°C. After the addition of methanol (10 mL) under 1 min of shaking, triphenylformazan (TPF) was extracted. Later, following sample collection, methanol was added to wash the test tube until the disappearance of red color. Later, all the filtrates were diluted until 100 mL, the absorbance values were measured through spectrophotometry at 485 nm, whereas the activity of dehydrogenase was displayed in the manner of mg TPF/g soil/h.

Peruccia et al.’s method [26] was utilized to determine the activity of polyphenol oxidase (EC 1.10.3.1). In brief, 1 g fresh soil was blended sufficiently with 3 mL reagent solution (acquired through blending 1.5 mL catechol solution (0.2 M) into 1.5 mL proline solution (0.2 M) together with 2 mL of the 0.1 M phosphate buffer (pH 6.5)). Then, after 10 min incubation of the resultant mixture under 30°C, 5 ml ethanol was added on the ice bath, followed by 4 min centrifugation at 5000 g under 4°C. Later, the supernatant absorbance value was determined at 525 nm. The soil- and catechol-free assays were performed at the same time as controls. The activities of enzymes were presented in the manner of μmols catechol oxidized/10 min/g soil (based on dry weight).

### Grain yield

At the physiological maturity stage, an area of 130 m^2^ (10 rows, 10 m long) from different plots was harvested manually, and the grain yield was determined. The number of plants and ears was counted. Plants from the middle rows were then chosen for cob sampling. The kernel number per ear was calculated, and the 100-kernel weight was determined after air-drying. Grain yield per hectare was expressed at 14% moisture content.

### Data analysis

SPSS16.0 (SPSS, Inc., Chicago, IL, US) was adopted for statistical analysis. Ducan’s Multiple Range Test (DMRT) along with the one-way ANOVA was performed to analyze each variable upon a significance threshold of 0.05. Minitab 16.0 (Minitab, State College, PA, USA) was utilized to compare and analyze PLFA profiles.

## Results

Straw incorporation has been considered to be the eco-friendly method to use straw byproducts, since it contains a large amount of nutrients [1]. As demonstrated in a number of studies, the effects of different maize straw incorporation modes in the soil differ because various applications can affect different biogeochemical processes in soil, such as soil microbial biomass carbon (MBC), microbial community structure, and enzyme activities [11]. This study selected chernozem soil to study the influence of different straw application modes on soil microbial compositions during the entire growth phase. The detailed results of the study are as follows:

### Soil microbial biomass carbon (MBC)

MBC represents the living SOC fraction and is considered as an estimate of biological activity in soil. Table 1 represents the results of soil microbial biomass carbon. In comparison to the control treatment (CK), MBC contents were increased with straw application (Table 1). In the 0–20 cm soil layer, the MBC content of PD treatment was the highest during the same growth period, which was 14.1%, 23.2%, and 56.3% higher than that of BD, M and CK, respectively, in 2018, and was 9.03%, 9.80%, and 18.5% higher than that of BD, M, and CK, respectively, in 2019. In the whole growth stage, the average soil MBC content of SF increased by 5.5%, 20.5%, and 37.0% (2018), 7.9%, 14.4%, and 36.0% (2019), respectively. In the 20–40 cm soil layer, the MBC contents of the same treatment first increased and then decreased after reaching the maximum value at the VT stage. The MBC content of the PD treatment was 389.7 and 384.6 mg kg^−1^ at the VT stage in 2018 and 2019, respectively, which were significantly higher than those in the BD, M, and CK treatments (*p* < 0.05).

**Table 1.** Effects of different straw returning mode on the soil microbial biomass carbon (mg/kg)

Soil layer	Year	Treatments	Growth Stage					
			V3	V6	V12	VT	R3	R6
0–20 cm	2018	PD	244.2 ± 10.1a	324.8 ± 8.3a	479.4 ± 11.2a	354.7 ± 9.9a	402.6 ± 9.3a	271.7 ± 9.9a
		BD	224.6 ± 9.8b	342.8 ± 9.4a	420.0 ± 10.7b	356.4 ± 10.3a	379.5 ± 10.2b	245.8 ± 11.4b
		M	185.4 ± 11.2 c	300.7 ± 8.9b	389.0 ± 10.4 c	287.1 ± 11.5b	313.7 ± 11.4 c	247.7 ± 10.6b
		CK	198.5 ± 9.7d	232.8 ± 9.9 c	306.6 ± 9.2d	270.3 ± 10.7 c	306.5 ± 10.7 c	201.4 ± 9.7 c
	2019	PD	193.5 ± 9.7a	289.3 ± 9.9a	389.4 ± 8.9a	332.7 ± 8.9a	361.6 ± 89.1a	212.1 ± 10.4b
		BD	180.9 ± 10.2b	267.8 ± 10.3b	365.0 ± 9.7b	305.1 ± 9.2b	319.1 ± 92.3b	210.3 ± 10.4b
		M	152.5 ± 9.4 c	275.9 ± 9.8b	330.2 ± 10.4 c	277.9 ± 10.3 c	297.8 ± 90.1b	220.5 ± 9.9a
		CK	154.7 ± 3.4 c	211.0 ± 7.9 c	285.9 ± 11.3d	234.5 ± 9.4d	265.1 ± 10.4 c	157.0 ± 10.5 c
20–40 cm	2018	PD	219.5 ± 11.0a	250.4 ± 9.9a	350.6 ± 10.5a	389.7 ± 11.3a	343.8 ± 10.4a	299.2 ± 10.5a
		BD	219.0 ± 10.9a	217.6 ± 9.4b	286.6 ± 11.4b	336.4 ± 11.2b	291.5 ± 9.9b	243.8 ± 9.8b
		M	175.3 ± 9.5b	202.2 ± 10.4 c	253.1 ± 10.6 c	319.4 ± 10.6 c	284.8 ± 10.4b	253.5 ± 12.4b
		CK	177.8 ± 9.5b	185.1 ± 10.6 c	248.2 ± 11.5 c	269.5 ± 9.5d	235.7 ± 11.3 c	180.4 ± 11.4 c
	2019	PD	182.5 ± 10.7a	260.4 ± 15.4a	330.6 ± 11.2a	384.6 ± 9.8a	283.8 ± 10.9a	219.2 ± 112.4a
		BD	169.0 ± 9.9b	187.6 ± 12.3b	266.6 ± 10.5b	306.4 ± 10.4b	261.5 ± 9.8b	213.8 ± 10.7a
		M	145.3 ± 12.6 c	182.2 ± 9.4b	233.1 ± 9.9 c	249.4 ± 9.9 c	214.8 ± 11.2 c	193.5 ± 9.8a
		CK	147.8 ± 12.9 c	155.1 ± 11.5 c	198.2 ± 10.4d	239.5 ± 10.4 c	205.7 ± 10.2d	150.4 ± 12.3b

Note: Values followed by different letters in the same column are significantly different at 5% significance level.

### Phospholipid fatty acid analyses (PLFA)

PLFA analysis is becoming a popular method for assessing microbial community structure in soils and estimating microbial biomass. In particular, PLFA microbial group ratios are generally used as standards for soil health interpretation and assessment. In this study, PLFA was significantly affected by the levels of straw application. In comparison to the control treatment (CK), the PLFAs of bacteria, actinomycetes and fungi were significantly higher with the straw application treatments (p < 0.05; Tables 2 and 3). In the 0–20 cm soil layer (Table 2), the average PLFAs of bacteria, actinomycetes and fungi in the whole growth stage were ranked in the order: PD (56.5,10.6,6.5 nmol g^−1^)>BD (51.8,10.3,5.9 nmol g^−1^)>M (41.6,8.3,4.4 nmol g^−1^)>CK

**Table 2.** Soil PLFAs variation of microbial groups under different treatments (0–20 cm)

Year	PLFA	Treatments	Growth Stage					
			V3	V6	V12	VT	R3	R6
2018	Bacterial PLFAs	PD	45.4 ± 1.3a	45.9 ± 0.3a	71.0 ± 1.6a	64.0 ± 2.3a	68.0 ± 2.8a	45.4 ± 3.5a
		BD	41.2 ± 2.1a	45.8 ± 0.5a	62.3 ± 1.3b	55.0 ± 2.8bc	60.0 ± 2.5b	46.5 ± 3.8a
		M	23.0 ± 1.0b	34.0 ± 0.6b	46.5 ± 1.0 c	51.4 ± 1.4b	52.0 ± 1.8 c	43.0 ± 4.1b
		CK	20.1 ± 0.9b	30.0 ± 1.1b	42.4 ± 2.1 c	48.7 ± 1.6 c	45.0 ± 3.1d	40.0 ± 3.6b
	Actinomycetic PLFAs	PD	12.23 ± 2.1a	12.96 ± 1.9a	10.94 ± 0.2b	9.28 ± 0.5a	9.16 ± 0.4a	8.76 ± 0.5a
		BD	6.84 ± 0.3b	8.92 ± 1.4b	10.61 ± 0.4b	8.84 ± 0.4a	9.47 ± 0.5a	6.98 ± 0.3bc
		M	6.32 ± 0.5b	7.67 ± 0.4bc	11.26 ± 0.3a	8.96 ± 0.4a	8.26 ± 1.4b	7.26 ± 0.6b
		CK	5.31 ± 0.3 c	6.22 ± 0.6 c	10.86 ± 1.0b	9.26 ± 0.5a	8.54 ± 0.5b	8.22 ± 0.1a
	Fungal PLFAs	PD	4.72 ± 0.2a	4.99± a0.4	7.29 ± 0.4a	7.96 ± 1.1a	7.02 ± 1.1a	6.96 ± 1.2a
		BD	3.72 ± 0.3b	5.06 ± 0.3a	6.04 ± 0.6b	6.65 ± 0.4b	5.01 ± 0.9b	5.43 ± 0.5b
		M	3.28 ± 0.4 c	3.94 ± 0.9b	4.42 ± 0.1 c	5.28 ± 0.5 c	5.05 ± 0.4b	4.52 ± 0.8 c
		CK	2.18 ± 0.6d	2.99 ± 0.1 c	4.29 ± 0.6 c	4.43 ± 0.6d	4.02 ± 0.5 c	3.66 ± 0.3d
2019	Bacterial PLFAs	PD	22.15 ± 2.0a	49.14 ± 1.3a	62.11 ± 1.5a	47.47 ± 3.1a	55.01 ± 1.5a	45.27 ± 1.0a
		BD	21.37 ± 1.8a	38.06 ± 1.1b	51.48 ± 1.3b	42.03 ± 0.4b	55.2.±0.5a	44.08 ± 0.4a
		M	20.44 ± 0.4b	30.84 ± 0.9 c	40.74 ± 0.9 c	40.03 ± 2.2b	25.07 ± 1.3 c	33.26 ± 0.9b
		CK	19.12 ± 0.7b	28.92 ± 2.0 c	39.32 ± 0.4 c	40.94 ± 1.6b	30.41 ± 2.0b	22.90 ± 1.1 c
	Actinomycetic PLFAs	PD	5.62 ± 0.5a	7.42 ± 0.4a	7.71 ± 0.4a	7.31 ± 0.1a	6.4 ± 0.3a	4.2 ± 0.4b
		BD	4.83 ± 0.9 c	7.71 ± 0.2a	7.33 ± 0.3a	7.03 ± 0.3 c	5.1 ± 0.6b	4.1 ± 0.3b
		M	5.11 ± 0.3b	7.23 ± 0.5ab	6.83 ± 0.2b	7.9 ± 0.1a	5.7 ± 0.1b	5.3 ± 0.3a
		CK	5.61 ± 0.5a	6.70 ± 0.3b	6.68 ± 0.5b	7.4 ± 0.2b	6.4 ± 0.4a	4.2 ± 0.5b
	Fungal PLFAs	PD	4.67 ± 0.06a	4.44 ± 0.03a	7.01 ± 0.04a	7.45 ± 0.43a	6.87 ± 1.11a	6.44 ± 0.45a
		BD	4.12 ± 0.10b	4.52 ± 0.05a	6.34 ± 0.32b	6.78 ± 0.12b	5.34 ± 0.92b	5.33 ± 0.97b
		M	3.02 ± 0.03 c	4.34 ± 0.03a	4.72 ± 0.25 c	4.84 ± 0.26 c	3.53 ± 1.11 c	2.88 ± 0.04 c
		CK	2.30 ± 0.06d	2.56 ± 0.12b	2.85 ± 0.39d	3.65 ± 0.19d	2.53 ± 0.41d	2.34 ± 0.06 c

Note: Values followed by different letters in the same column are significantly different at 5% significance level.

**Table 3.** Soil PLFAs variation of microbial groups under different treatments (20–40 cm)

Year	PLFA	Treatments	Growth Stage					
			V3	V6	V12	VT	R3	R6
2018	Bacterial PLFAs	PD	24.0 ± 1.1a	28.0 ± 1.5b	45.2 ± 0.9a	56.0 ± 1.1a	46.0 ± 4.6a	43.2 ± 4.0a
		BD	20.1 ± 1.9a	31.0 ± 1.9a	40.3 ± 1.2b	45.0 ± 2.5b	39.0 ± 3.8b	40.3 ± 4.2a
		M	17.0 ± 0.4b	20.0 ± 0.4 c	29.0 ± 0.9 c	39.0 ± 1.4 c	35.0 ± 3.1 c	33.0 ± 3.9b
		CK	16.5 ± 0.9b	19.0 ± 0.8 c	25.0 ± 1.8d	32.0 ± 1.6d	27.4 ± 2.9d	23.1 ± 4.0 c
	Actinomycetic PLFAs	PD	4.53 ± 0.4a	5.43 ± 0.4a	6.18 ± 0.9a	6.94 ± 0.6a	6.23 ± 0.4a	5.19 ± 0.5a
		BD	4.08 ± 0.5b	4.04 ± 0.1b	4.68 ± 1.3b	6.12 ± 0.4b	5.30 ± 0.2b	3.03 ± 0.6b
		M	3.10 ± 0.1 c	2.74 ± 1.0 c	4.46 ± 1.1b	4.51 ± 0.3 c	3.06 ± 1.1 c	3.02 ± 1.1b
		CK	2.07 ± 0.2d	2.22 ± 0.6 c	2.26 ± 0.5 c	3.71 ± 0.4d	1.85 ± 0.6d	1.98 ± 0.3 c
	Fungal PLFAs	PD	3.05 ± 0.4a	3.56 ± 0.4a	6.31 ± 0.4a	6.71 ± 0.3a	5.78 ± 0.3a	5.61 ± 0.6a
		BD	2.20 ± 0.1b	2.99 ± 0.6b	4.55 ± 0.2b	4.75 ± 0.6b	4.97 ± 0.5b	4.40 ± 0.1b
		M	1.44 ± 0.2 c	1.79 ± 0.3d	2.72 ± 0.4 c	3.41 ± 0.4 c	3.40 ± 0.1 c	3.12 ± 0.5 c
		CK	1.74 ± 0.1 c	2.09 ± 0.6 c	2.17 ± 0.6 c	2.29 ± 0.9d	1.08 ± 0.3d	1.01 ± 0.3d
2019	Bacterial PLFAs	PD	20.13 ± 0.8a	18.00 ± 0.9a	34.30 ± 1.2a	49.00 ± 1.7a	43.56 ± 2.6a	42.05 ± 1.5a
		BD	15.07 ± 2.4b	19.00 ± 1.3a	32.02 ± 1.0a	46.56 ± 1.2b	40.68 ± 1.4a	39.36 ± 1.2b
		M	12.64 ± 1.1 c	16.00 ± 2.1b	25.59 ± 2.4b	39.49 ± 0.5a	35.65 ± 1.9b	35.62 ± 0.6 c
		CK	10.54 ± 0.8 c	16.90 ± 1.4b	24.59 ± 0.7b	36.19 ± 1.1 c	31.71 ± 0.3 c	23.35 ± 0.9d
	Actinomycetic PLFAs	PD	2.15 ± 0.4b	4.01 ± 0.4b	5.61 ± 0.1a	5.97 ± 0.1a	5.14 ± 0.3a	3.94 ± 0.6a
		BD	2.61 ± 0.9a	3.97 ± 0.5b	5.08 ± 0.5b	5.76 ± 0.1a	5.37 ± 0.6a	3.46 ± 0.3b
		M	2.15 ± 0.3b	4.25 ± 0.1a	4.91 ± 0.4b	5.25 ± 0.4b	4.62 ± 0.4b	2.64 ± 0.4 c
		CK	2.01 ± 0.7 c	4.06 ± 0.1b	4.05 ± 0.2 c	4.92 ± 0.2b	4.34 ± 0.6b	2.23 ± 0.3d
	Fungal PLFAs	PD	2.42 ± 0.05a	2.32 ± 0.11a	3.65 ± 0.40a	3.85 ± 0.66a	4.61 ± 0.61a	4.16 ± 0.06a
		BD	1.83 ± 0.43b	2.05 ± 0.14b	2.89 ± 0.33b	2.82 ± 0.37b	3.92 ± 0.43b	3.35 ± 0.04b
		M	1.16 ± 0.04b	1.41 ± 0.23 c	2.08 ± 0.24 c	2.53 ± 0.41b	3.25 ± 0.06 c	3.06 ± 0.05 c
		CK	1.01 ± 0.01 c	1.38 ± 0.04 c	1.93 ± 0.09 c	2.02 ± 0.36 c	2.36 ± 0.02d	2.06 ± 0.02d

Note: Values followed by different letters in the same column are significantly different at 5% significance level.

(38.3,8.1,3.6 nmol g^−1^) in 2018. No significant differences (*p* < 0.05) in average bacterial, actinomycetes and fungi PLFAs between PD and BD were observed, which were significantly higher than CK (*p* < 0.05).

Compared to the 0–20 cm soil layer, the mean PLFAs of bacteria, actinomycetes, and fungi in the whole growth stage were lower in 20–40 cm soil layer (Table 3). The mean PLFAs of bacteria, actinomycetes and fungi in the whole growth stage were ranked in the order: PD (40.3,5.8,5.2 nmol g^−1^)>BD (35.8,4.5,4.0 nmol g^−1^)>M (28.8,3.5,2.6 nmol g^−1^)>CK(23.7,2.3,1.7 nmol g^−1^) in 2018, PD(34.5,4.5,3.5 nmol g^−1^)>BD(32.1,4.38,2.81 nmol g^−1^)>M(27.5,3.97,2.25 nmol g^−1^)>CK(22.3,3.6,1.79 nmol g^−1^) in 2019.

### Soil enzyme activities

Soil enzymes activity is an essential ecosystem process as enzymes catalyze countless reactions in soil that have biogeochemical significance. These soil enzymes, such as invertase, urease, and others, decompose dead plants and complex forms of organic matter into accessible nutrient elements, i.e. C-, N-, and P. In this study, the straw application influenced the activities of urease, invertase, dehydrogenase and polyphenoloxidase (Tables 4 and 5). As the growth period progressed, the soil urease activities in the 0–20 cm layer gradually increased and began to decline after the R3 period in both years (Table 4). In the 0–20 cm soil layer (Table 4), no significantly differences (*p* < 0.05) in the mean urease and invertase activities in the whole growth stage between PD and BD were observed, which were both significantly higher than M (*p* < 0.05) in both years. The mean activities of polyphenoloxidase in PD treatment at the growth stage was significantly higher than that of BD, M, and CK, which were increased by 10.1%, 46.5% and 110%, respectively, in 2018, and were increased by 20.2%,38.7% and 99.9%, respectively, in 2019.

**Table 4.** Soil enzyme activities under different treatments (0–20 cm)

Year	Soil enzyme	Treatments	Growth Stage					
			V3	V6	V12	VT	R3	R6
2018	Urease	PD	0.35 ± 0.02a	0.32 ± 0.02b	0.44 ± 0.03b	0.52 ± 0.01a	0.62 ± 0.03a	0.49 ± 0.02a
		BD	0.39 ± 0.01a	0.45 ± 0.04a	0.54 ± 0.02a	0.56 ± 0.02a	0.58 ± 0.01a	0.37 ± 0.03b
		M	0.19 ± 0.02 c	0.31 ± 0.03b	0.31 ± 0.01 c	0.41 ± 0.01b	0.46 ± 0.02b	0.26 ± 0.02 c
		CK	0.28 ± 0.03b	0.29 ± 0.01b	0.31 ± 0.03 c	0.35 ± 0.03 c	0.39 ± 0.03 c	0.16 ± 0.01d
	Invertase	PD	7.89 ± 0.62a	8.34 ± 0.34a	8.66 ± 1.01b	11.36 ± 1.00a	9.75 ± 0.38a	8.12 ± 0.34b
		BD	7.48 ± 0.34a	8.94 ± 0.45a	10.03 ± 0.89a	10.57 ± 1.14b	9.52 ± 0.75a	8.99 ± 0.51a
		M	6.04 ± 0.51b	6.46 ± 0.38b	8.33 ± 0.34b	8.45 ± 0.54 c	7.45 ± 0.82b	5.50 ± 0.68 c
		CK	5.06 ± 0.46 c	5.73 ± 0.49 c	6.64 ± 0.48 c	7.27 ± 0.56d	5.78 ± 0.36 c	5.40 ± 0.36 c
	Dehydrogenase	PD	4.21 ± 0.61a	4.21 ± 0.74b	5.65 ± 0.83b	6.35 ± 0.43a	5.39 ± 0.87a	4.24 ± 0.85a
		BD	4.35 ± 0.66a	6.35 ± 0.72a	6.73 ± 0.52a	6.02 ± 0.91a	5.92 ± 0.35a	4.10 ± 0.99a
		M	2.64 ± 0.55b	2.64 ± 0.66 c	3.90 ± 0.26 c	4.04 ± 0.61b	3.92 ± 0.62b	3.02 ± 0.75b
		CK	2.05 ± 0.61 c	2.35 ± 0.67 c	3.89 ± 0.77 c	3.01 ± 0.84 c	3.05 ± 0.69 c	2.69 ± 0.49 c
	Polyphenoloxidase	PD	3.01 ± 0.56b	4.18 ± 0.85a	3.73 ± 0.36b	4.96 ± 0.22a	4.66 ± 0.63a	2.94 ± 0.36a
		BD	3.18 ± 0.35a	3.16 ± 0.43b	4.15 ± 0.53a	4.06 ± 0.32b	4.06 ± 0.29b	2.71 ± 0.22a
		M	1.76 ± 0.32 c	2.26 ± 0.32 c	3.08 ± 0.72b	3.76 ± 0.11 c	3.06 ± 0.27 c	2.11 ± 0.51b
		CK	1.21 ± 0.56d	1.54 ± 0.32d	2.06 ± 0.47 c	2.46 ± 0.51d	2.35 ± 0.52d	1.58 ± 0.22 c
2019	Urease	PD	0.36 ± 0.02a	0.35 ± 0.02a	0.41 ± 0.03a	0.56 ± 0.02a	0.61 ± 0.03a	0.39 ± 0.04a
		BD	0.27 ± 0.03b	0.37 ± 0.01a	0.49 ± 0.02a	0.54 ± 0.03a	0.57 ± 0.04a	0.37 ± 0.03a
		M	0.21 ± 0.01 c	0.24 ± 0.04b	0.29 ± 0.03b	0.40 ± 0.01b	0.45 ± 0.02b	0.26 ± 0.02b
		CK	0.19 ± 0.01 c	0.21 ± 0.02b	0.21 ± 0.01b	0.30 ± 0.05 c	0.25 ± 0.05 c	0.21 ± 0.03b
	Invertase	PD	7.11 ± 0.43a	7.45 ± 1.34a	7.52 ± 0.47a	9.12 ± 0.45a	7.16 ± 0.30b	6.04 ± 0.55a
		BD	7.16 ± 0.62a	7.23 ± 0.89a	9.11 ± 0.28a	8.16 ± 0.66b	8.13 ± 0.31a	6.18 ± 0.37a
		M	4.56 ± 0.89b	5.03 ± 0.67b	6.02 ± 0.91b	6.83 ± 0.34 c	5.15 ± 0.34 c	5.09 ± 0.41b
		CK	4.15 ± 1.01b	4.22 ± 0.44b	3.35 ± 0.66 c	4.88 ± 0.21d	4.02 ± 0.56d	4.00 ± 0.34 c
	Dehydrogenase	PD	3.54 ± 0.75a	4.41 ± 072a	6.37 ± 0.73a	5.85 ± 0.68a	5.42 ± 0.67a	4.04 ± 0.73a
		BD	3.35 ± 0.70a	4.65 ± 0.69a	4.95 ± 032b	5.12 ± 0.63b	4.87 ± 0.61b	3.60 ± 0.92b
		M	2.14 ± 0.10b	2.94 ± 0.62b	3.40 ± 0.62 c	3.94 ± 0.58 c	3.42 ± 0.57 c	2.52 ± 0.82 c
		CK	1.91 ± 0.58b	2.75 ± 0.85b	2.69 ± 0.72d	3.51 ± 0.41d	2.55 ± 0.95d	2.19 ± 098 c
	Polyphenoloxidase	PD	2.47 ± 0.51a	3.83 ± 0.57a	3.60 ± 0.66a	4.25 ± 0.17a	3.29 ± 0.16a	2.81 ± 0.54a
		BD	1.87 ± 0.60 c	2.66 ± 0.65b	3.17 ± 0.18b	3.41 ± 0.18b	3.04 ± 0.27a	2.70 ± 0.55a
		M	2.00 ± 0.75b	2.09 ± 0.19 c	2.79 ± 1.00 c	3.03 ± 1.06 c	2.66 ± 0.95b	2.03 ± 0.90b
		CK	1.08 ± 0.79d	1.91 ± 0.18 c	2.05 ± 0.87d	2.02 ± 1.14d	1.67 ± 098 c	1.40 ± 0.81 c

Note: Values followed by different letters in the same column are significantly different at 5% significance level.

**Table 5.** Soil enzyme activities under different treatments (20–40 cm)

Year	Soil enzyme	Treatments	Growth Stage					
			V3	V6	V12	VT	R3	R6
2018	Urease	PD	0.32 ± 0.02a	0.41 ± 0.02a	0.41 ± 0.02a	0.49 ± 0.03a	0.41 ± 0.02a	0.37 ± 002a
		BD	0.23 ± 0.02b	0.39 ± 0.03a	0.38 ± 0.03a	0.39 ± 0.02b	0.29 ± 0.01b	0.27 ± 0.03b
		M	0.22 ± 0.03b	0.21 ± 0.01b	0.18 ± 0.01b	0.24 ± 0.04 c	0.27 ± 0.03b	0.24 ± 0.10b
		CK	0.17 ± 0.01 c	0.18 ± 0.02b	0.15 ± 0.03b	0.21 ± 0.01 c	0.21 ± 0.01 c	0.17 ± 0.04 c
	Invertase	PD	4.59 ± 0.78a	4.90 ± 0.49a	5.34 ± 0.35a	8.87 ± 1.04a	6.53 ± 0.55a	6.32 ± 0.53a
		BD	3.60 ± 0.65b	5.01 ± 0.44a	5.28 ± 0.32a	6.32 ± 0.63b	5.13 ± 0.42b	4.87 ± 0.62b
		M	3.19 ± 0.66 c	3.46 ± 0.83b	3.55 ± 0.54b	4.89 ± 0.24 c	5.04 ± 0.37b	3.02 ± 0.46 c
		CK	2.99 ± 0.73 c	3.24 ± 0.25b	2.79 ± 0.21 c	3.98 ± 0.15d	2.39 ± 0.41 c	2.29 ± 0.28d
	Dehydrogenase	PD	3.19 ± 0.60a	3.87 ± 0.58a	4.85 ± 0.63a	5.14 ± 0.75a	4.33 ± 0.72a	3.99 ± 0.73a
		BD	2.96 ± 055a	3.23 ± 0.52b	3.74 ± 0.25b	4.58 ± 0.70b	3.59 ± 0.60b	3.17 ± 0.32b
		M	1.80 ± 0.50b	2.31 ± 0.51 c	3.59 ± 0.24b	3.39 ± 0.61 c	2.44 ± 0.62 c	2.11 ± 0.72 c
		CK	1.71 ± 0.94b	2.02 ± 0.42d	1.73 ± 0.72 c	2.39 ± 0.58d	2.01 ± 0.51d	1.45 ± 0.21d
	Polyphenoloxidase	PD	2.38 ± 028a	3.42 ± 0.53a	3.58 ± 0.31a	4.07 ± 0.39a	3.36 ± 0.35a	2.38 ± 0.42a
		BD	1.44 ± 0.19b	2.39 ± 0.31b	2.41 ± 0.31b	3.39 ± 0.46b	2.42 ± 0.41b	0.41 ± 0.53 c
		M	0.50 ± 0.39 c	2.26 ± 0.45b	2.46 ± 0.54b	2.07 ± 0.58 c	2.43 ± 0.56b	1.40 ± 0.37b
		CK	0.51 ± 0.35 c	0.77 ± 0.63 c	1.45 ± 0.52 c	1.43 ± 0.62d	0.74 ± 0.52 c	0.41 ± 0.49 c
2019	Urease	PD	0.22 ± 0.01a	0.34 ± 0.01a	0.38 ± 0.02a	0.49 ± 002a	0.47 ± 0.02a	0.37 ± 0.03a
		BD	0.22 ± 0.03a	0.38 ± 0.02a	0.34 ± 0.02b	0.40 ± 0.03b	0.39 ± 0.03b	0.37 ± 0.01a
		M	0.20 ± 0.02a	0.23 ± 0.04b	0.23 ± 0.04d	0.29 ± 0.01 c	0.24 ± 0.01 c	0.27 ± 0.05b
		CK	0.14 ± 0.01b	0.25 ± 0.01b	0.27 ± 0.01 c	0.30 ± 0.02 c	0.16 ± 002d	0.12 ± 0.01 c
	Invertase	PD	4.53 ± 0.35a	5.43 ± 0.37a	6.18 ± 1.06a	6.94 ± 0.56a	6.23 ± 0.46a	5.19 ± 1.03a
		BD	4.08 ± 0.26b	4.04 ± 0.44b	4.68 ± 0.78b	6.12 ± 0.72b	5.30 ± 0.49b	3.03 ± 0.78b
		M	3.10 ± 0.72 c	2.74 ± 0.51 c	4.46 ± 0.59b	4.51 ± 0.33 c	3.06 ± 0.61 c	3.02 ± 0.95b
		CK	2.07 ± 0.35d	2.22 ± 0.29d	2.26 ± 0.35 c	3.71 ± 0.62d	1.85 ± 0.72d	1.98 ± 0.47 c
	Dehydrogenase	PD	3.19 ± 0.65a	4.57 ± 0.61a	4.95 ± 0.27a	5.44 ± 0.84a	4.33 ± 0.49a	3.99 ± 0.76a
		BD	2.16 ± 0.64b	2.63 ± 0.55b	3.24 ± 0.21b	3.12 ± 0.35b	3.24 ± 0.44b	3.02 ± 0.57b
		M	1.80 ± 0.53 c	2.16 ± 0.47 c	2.92 ± 0.20b	2.35 ± 0.30 c	2.95 ± 0.42 c	2.01 ± 0.49 c
		CK	1.22 ± 0.64d	1.64 ± 0.63d	1.66 ± 0.34 c	2.25 ± 0.73 c	2.01 ± 0.94d	2.01 ± 0.84 c
	Polyphenoloxidase	PD	2.27 ± 0.30a	3.67 ± 0.32a	3.74 ± 0.28a	4.11 ± 0.40a	3.67 ± 0.24a	3.19 ± 0.12a
		BD	1.47 ± 0.32b	2.75 ± 0.35b	3.23 ± 0.36b	3.79 ± 0.49a	3.28 ± 0.50b	2.75 ± 0.30b
		M	1.53 ± 0.36b	2.17 ± 0.50 c	1.97 ± 0.53 c	3.11 ± 0.63b	2.17 ± 0.51 c	1.49 ± 0.15 c
		CK	0.47 ± 0.45 c	1.05 ± 0.53d	1.69 ± 0.37 c	2.08 ± 0.89 c	1.18 ± 0.65d	0.92 ± 0.16d

Note: Values followed by different letters in the same column are significantly different at 5% significance level.

Under the same growth period, the polyphenoloxidase activity of 20–40 cm subsurface soil was lower than that of 0–20 cm top soil (Table 5). Compared to the control, the mean urease activities of PD, BD, and M treatments were increased by 121.1%, 78.9%, and 24.8%, respectively, in 2018, and were increased by 83.1%, 69.4%, and 17.7%, respectively, in 2019. The activities of soil dehydrogenase and polyphenoloxidase in PD treatment were also higher significantly (*p* < 0.05) than those of BD, M, and CK.

### Maize yield

**Figure 2.**
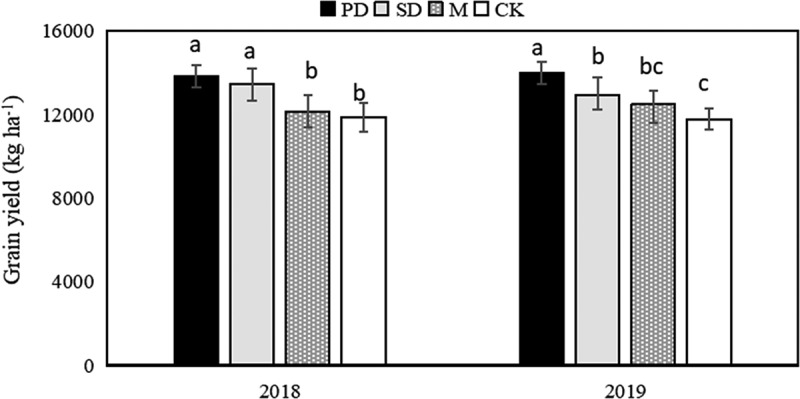
Grain yield in the different treatments. Values followed by different letters in the same column are significantly different at 5% significance level

The grain yield of PD treatment was higher than that of SD, M, and CK (Figure 2). There was no significant difference between PD and SD treatments, which were both significantly higher than those of M and CK in 2018. However, there was a significant difference between PD and SD treatments in 2019 (Figure 2), and the yield of the PD was 8.46%, 12.2%, and 18.9% higher than SD, M , and CK treatment, respectively. In both years, the mean yield of the PD and SD treatment was 17.7% and 11.5% higher than that of the control and M treatment, respectively. There was no significant difference between M treatment and CK.

## Discussion

Microorganisms participate in biochemical cycles and constitute the living component of soil organic matter, playing a vital role in nutrient transformation and conservation processes in cropland ecosystems. Soil microbial biomass carbon is a sensitive index to reflect the total amount of microorganisms and to evaluate the availability of soil nutrients and changes in soil microbial status [27,28]. Changes in soil environment will lead to changes in soil microbial biomass carbon [29]. Phospholipid fatty acid profile is an important monitoring index of soil microbial population change, which can reflect the difference in microbial biomass and community structure. The combination of the two can more comprehensively reflect the soil microbial biomass [30]. In the western part of Jilin province, local farmers use a variety of ways to return straw to the field. In this study, we examined some of those techniques for the first time in chernozem soil to check the effect of different straw application modes on soil microbial biomass carbon (C), microbial community structure, and enzyme activities during the entire growth phase.

Application of corn straw is a method used frequently to improve the soil quality. Crop straw decomposition is an effective way of soil carbon cycle and nutrient regeneration and plant residue can regulate the basic biological functions of soil [17]. Straw application is recognized to be directly related to MBC. It is discovered in studies that straw incorporation, in particular the straw burial, elevates sources of soil carbon and offers nutritional elements to facilitate the propagation of microorganisms [31]. Previous studies have shown that some allelopathic compounds are released with the decomposition of crop straw [32], which can also influence or alter soil microbial community diversity [33,34]. The results differ according to the soil types, straw sizes, temperature, moisture, fertilizer, and cultivation systems.

In this study, by comparison to the treatment without straw, soil microbial biomass carbon and main characteristic fatty acid content were influenced significantly and positively by the application of corn straw, whether it is 0–20 cm topsoil or 20–40 cm sub-topsoil soil. Compared with different patterns, the effect of PD treatment was significant. Some studies have shown that the decomposition rate of straw deep buried treatment is significantly higher than that of straw mulching treatment, which can effectively improve the soil plow bottom, increase the number of soil beneficial microorganisms, and the deep-buried straw inter-layer increased the organic matter, providing rich C sources for microorganism growth [15].

Compared with different soil layers, the MBC, the PLFAs of bacteria, actinomycetes and fungi in 20–40 cm soil layer were lower significantly (p < 0.05) than that of 0–20 cm in the early stage of maize. With the growth of maize, the microbial biomass carbon content of sub-plow layer increased rapidly. The improvement of the deep soil layer is beneficial to the development of roots. In turn, the penetration of roots can also enhance the soil structure [35]. Additionally, crop roots can release different types of soluble sugars, amino acids, or secondary metabolites (allelochemicals) to be used by soil microbial communities [36,37], which are very helpful for underground microecosystems and aboveground crops.

The activities of enzymes are greatly dependent on the growing roots together with the active microbiomes [38] and they show high sensitivity and can rapidly respond to the changes or disturbance or the increase in organic matters [39]. The polyphenol oxidase exerts an important part in the cycling of aromatic compounds, whereas the dehydrogenase serves as the soil bioactivity index. Invertase can catalyze sucrose hydrolysis into fructose and glucose, which is associated with the microbial biomass in soil [40]. Urease exerts an important part during urea hydrolysis, and its efficiency can be reduced within a soil ecosystem [41]. An improved understanding of the role of these four enzymes in improving soil quality would allow us to plan more effective changes in maize ecosystems.

Our results further indicated that the activities of urease, invertase, dehydrogenase and polyphenoloxidase were all altered by the straw application. In the 0–20 cm soil layer, no significant differences (*p* > 0.05) in the mean urease and invertase activities in the whole growth stage between PD and BD were observed, which were both significantly higher than M (*p* < 0.05) in both years. However, in the 20–40 cm soil layer, the mean activities of four soil enzymes in the PD treatment were higher significantly (*p* < 0.05) than those of other treatments. In deep soil layers, buried straw can release more nutrients during decomposition, which would stimulate the deep roots and result in different kinds of metabolites production [42,43], stimulating soil microbial biomass, and leading to fluctuations in the enzyme activity of the soil layer. And polyphenol oxidase may have been associated with the release of phenolic compounds – a type of secondary metabolite – by the plant roots during straw decomposition [44].

In recent years, maize straw biomass has dramatically increased with increases in planting density and yield, and straw incorporation has been widely applied to enhance fertilization and soil quality and to promote the development of sustainable agriculture in China. Our results indicated that the application of straw had positive effects on soil microorganisms to some extent, but there was different response to the different straw returning pattern. The straw incorporation rate, straw size, fertilizer quantity, and duration of the straw treatment influence crop yields [45]. Straw incorporation has a long-term effect on maize yield, and shorter durations of straw incorporation might lead to a relatively low yield response [46,47]. Our field experiment was carried out in the seri-dried area in north-east China, and we have long time to attempt different patterns in different regions to return the straw to the field. Although the farmland is gradually concentrated and managed by some farmers’ cooperative organizations, there is still a household planting mode. Therefore, there are many different ways of straw returning, and farmers have invented many machines themselves. As agricultural researchers, we need to analyze the influences of different ways of returning straw on soil property, quality, and even yield.

## Conclusion

In the present study, different straw incorporation methods were used to observe the influence on soil microbial community structure, soil microbial biomass carbon content, and enzymatic activities. The results of this showed no significant increase in the soil microorganisms by straw mulching method. However, straw deep plowing and returning (PD treatment) method effectively improved the soil MBC, the PLFAs of bacteria, actinomycetes and fungi, the activities of urease, invertase, dehydrogenase and polyphenoloxidase, during the grain yield. Notably, in other experimental sites of our team, the effect of straw mulching was significant, in terms of increased soil microorganisms. Thus, in the actual field production, we should adjust measures according to the characteristics of climate and soil in different regions, and adopt the appropriate way of straw returning to the field to achieve a win-win situation of soil protection and yield increase.

## Disclosure of potential conflicts of interest

No potential conflict of interest was reported by the author(s).

## Article highlights

- Soil microorganisms were not increased by straw mulching
- PD treatment effectively improve soil MBC, PLFAs and enzymes activity.
- The effects of subsoil (20-40 cm) were more obvious than those of topsoil.
- The mean yield of PD treatment was higher than SD, M and CK.
